# Mechanistic insights into the recognition of 5-methylcytosine oxidation derivatives by the SUVH5 SRA domain

**DOI:** 10.1038/srep20161

**Published:** 2016-02-04

**Authors:** Eerappa Rajakumara, Naveen Kumar Nakarakanti, M. Angel Nivya, Mutyala Satish

**Affiliations:** 1 Department of Biotechnology, Indian Institute of Technology Hyderabad, Kandi, Sangareddy, 502285, Telangana, India

## Abstract

5-Methylcytosine (5 mC) is associated with epigenetic gene silencing in mammals and plants. 5 mC is consecutively oxidized to 5-hydroxymethylcytosine (5 hmC), 5-formylcytosine (5fC) and 5-carboxylcytosine (5caC) by ten-eleven translocation enzymes. We performed binding and structural studies to investigate the molecular basis of the recognition of the 5 mC oxidation derivatives in the context of a CG sequence by the SET- and RING-associated domain (SRA) of the SUVH5 protein (SUVH5 SRA). Using calorimetric measurements, we demonstrate that the SRA domain binds to the hydroxymethylated CG (5hmCG) DNA duplex in a similar manner to methylated CG (5mCG). Interestingly, the SUVH5 SRA domain exhibits weaker affinity towards carboxylated CG (5caCG) and formylated CG (5fCG). We report the 2.6 Å resolution crystal structure of the SUVH5 SRA domain in a complex with fully hydroxymethyl-CG and demonstrate a dual flip-out mechanism, whereby the symmetrical 5hmCs are simultaneously extruded from the partner strands of the DNA duplex and are positioned within the binding pockets of individual SRA domains. The hydroxyl group of 5hmC establishes both intra- and intermolecular interactions in the binding pocket. Collectively, we show that SUVH5 SRA recognizes 5hmC in a similar manner to 5 mC, but exhibits weaker affinity towards 5 hmC oxidation derivatives.

5-methylcytosine (5 mC) is an epigenetic mark in the genomic DNA that plays important roles in modulating transcriptional activities, genomic imprinting and suppressing transposable elements in most eukaryotic organisms. In mammals, DNA methylation is predominantly found in the context of a symmetric CG (5 mCG)^1^,^2^, whereas in plants, DNA methylation usually occurs in all sequence contexts: symmetric CG and CHG (5 mCHG) (where H = A, T, or C), and asymmetric CHH (5 mCHH)^3^. In a symmetric sequence, 5 mC can be fully methylated (fully-5 mCG or fully-5 mCHG) (Fig. 1B) or hemi-methylated (hemi-5 mCG or hemi-5 mCHG). The establishment and maintenance of 5 mC is required for both the inheritance and transmission of epigenetic signals from the mother cell to the daughter cell. The role of the SET- and RING-associated (SRA) domain proteins in the establishment and/or maintenance of 5 mC in both plants and mammals was reported previously^3^.

In *Arabidopsis*, the SRA domain of the SU(VAR)3-9 HOMOLOG (SUVH) histone methyltransferase recognizes 5 mC (mC) in different sequence contexts and methylation statuses. The methylated CG status can be hemi-5 mCG (5′mCG3′/3′GC5′) or fully-5 mCG (5′mCG3′/3′GmC5′). Similarly, the methylated CHG status can be hemi-5 mCHG (5′mCHG3′/3′GDC5′; H is non-G whereas D is the complementary base of H) or fully-5 mCHG (5′mCHG3′/3′GDmC5′)^4^,^5^. The recognition of methylated DNA is required for the maintenance of the repressive marks 5 mC and histone H3 lysine dimethylation (H3K9me2), and for silencing the transposable elements and gene transcription in plants^3^,^6^. UHRF1 is a RING finger-associated mammalian SRA domain protein that is required to maintain 5 mC in the CG context^7^,^8^. UHRF1 SRA specifically recognizes hemi-5 mCG sites^8^,^9^,^10^,^11^,^12^, which are the product of semi-conservative DNA replication. Furthermore, by recruiting maintenance DNA methyltransferase (DNMT1), UHRF1 SRA facilitates the restoration of hemi-5 mCG to fully-5 mCG after each round of DNA replication^7^,^8^ in mammals.

Recent reports have revealed that 5 mC is oxidized to 5-hydroxymethylcytosine (5 hmC), 5-formylcytosine (5 fC), and 5-carboxylcytosine (5 caC) through three consecutive oxidation reactions catalysed by ten-eleven translocation (Tet) family enzymes in mammals^13^,^14^ (Fig. 1A). Although 5 mC oxidation derivatives are reported in plant genomic DNA in a tissue-specific pattern^15^, their origin is unknown. It is unlikely that these bases are generated by TET-mediated oxidation in plants. In mammals and plants, these modified bases may represent new epigenetic status in the genomic DNA or intermediates in the process of 5 mC demethylation^16^,^17^.

Despite the structural conservation in the SRA domain fold, the binding specificities of previously characterized SRA domains vary greatly, both in their recognition of 5 mC and 5 hmC and in their modification status^18^. The fully (Fig. 1B,C,E,F) and hemi-methylated statuses (Fig. 1D) are identified by the presence of two (one on each partner strands) and one (on one of the partner strands) modified base(s) (5 mC, 5 hmC, 5 fC or 5 caC), respectively, in symmetric (CG or CHG) sequence contexts. The SRA domain of UHRF1 (UHRF1 SRA) selectively recognizes 5 mC over 5 hmC^18^, and preferentially binds hemi-5 mCG over fully-5 mCG^8^,^18^. By contrast, the SRA domain of UHRF2 (UHRF2 SRA) preferentially binds 5 hmC over 5 mC, and fully-5 hmCG over hemi-5 hmCG^18^.

Previous structural studies have indicated that UHRF2 SRA recognizes hemi-5 hmCG through a dual flip-out of the 5 hmC and C bases, which is reminiscent of the recognition of hemi-5 mCG by the SUVH5-SRA domain^4^,^18^. Conversely, the crystal structures of UHRF1 bound to hemi-5 mCG and of SUVH4 bound to hemi-5 mCHG and 5 mCHH DNAs have revealed that only the 5 mC is flipped-out from the duplex DNA^10^,^19^. Surprisingly, the methyl-specific endonuclease McrBC from *E. coli*, which has a distinct fold from those of SRA domain proteins, recognizes the 5 mC bases from the partner strands through a dual flip-out mechanism^20^. Similarly, the DNA-free structure of the 5 hmC- and 5- glucosylhydroxymethylcytosine (5 ghmC)-specific endonuclease PvuRts1I indicated that its C-terminal SRA domain might accommodate a flipped out 5 hmC or 5 ghmC base^21^. Another 5 hmC- and 5 ghmC-specific restriction enzyme, AbaSI, recognizes intra-helical 5 hmC through an SRA-like domain^22^. However, there are no structural data available for the recognition of fully-5 hmCG by the SRA domain proteins.

To better understand the binding specificity of the SRA domains in general, and of SUVH5 in particular, the binding preferences of the SUVH5 SRA domain for 5 mC oxidation derivatives in the fully-CG modification status were determined. Our studies are the first to establish that SUVH5 SRA specifically binds 5 mC and 5 hmC bases, but exhibits weaker affinities towards 5 fC and 5 caC modified bases. The recognition of 5 hmC is supported by the structure of SUVH5 SRA in complex with fully-5 hmCG duplex DNA, which reveals the dual flip-out of 5 hmC bases on the partner strands for 5 hmC recognition. Importantly, this study also unearths the preference of SUVH5 SRA for fully-5 hmCG over hemi-5 hmCG, which supports our previous report showing preferential recognition of fully-5 mCG over hemi-5 mCG^4^.

## Results

### Calorimetric studies of the 5 hmC-binding specificity of the SUVH5 SRA domain

**Figure 1:**
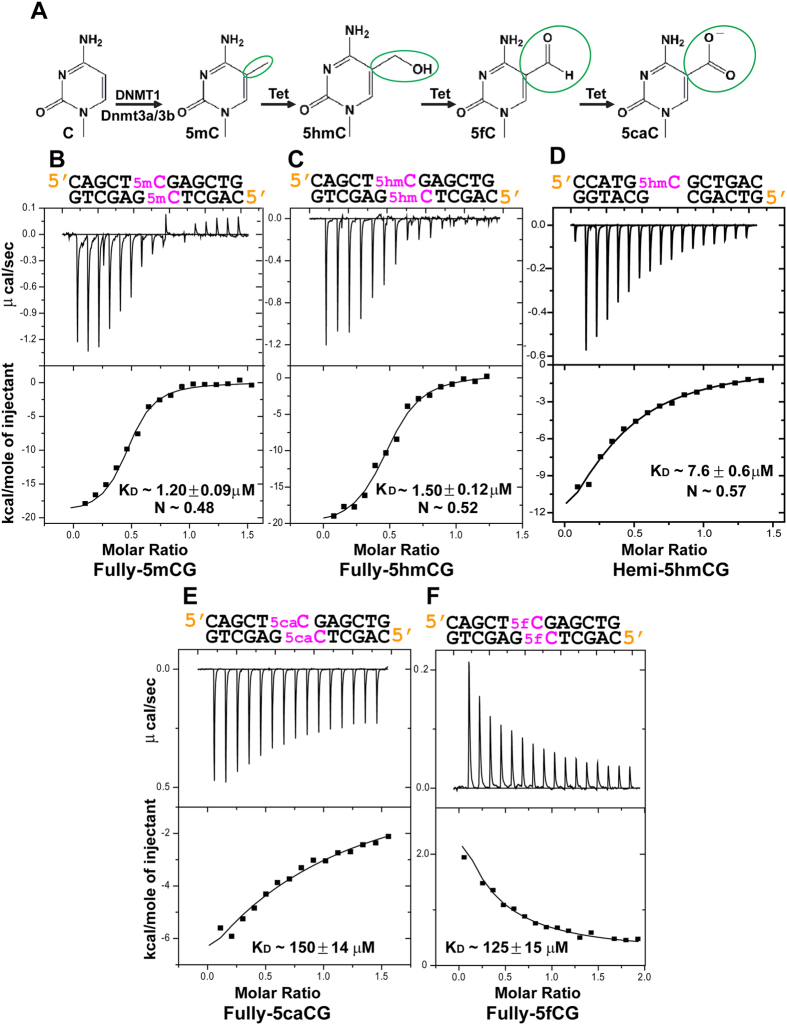
Binding of the SUVH5 SRA domain to the fully-5 mCG, fully-5 hmCG, hemi-5 hmCG, fully-5 caCG and fully-5 fCG-containing duplex DNAs. The thermodynamic data are provided in Table 1. (**A**) The flow-chart represents the methylation of cytosine by the DNMT1 and Dnmt3a/3b methyltransferases and the sequential oxidation of 5 mC to 5 caC by the Tet family of enzymes in mammals. (**B**) ITC measurements of the binding of the SUVH5 SRA domain to the fully-5 mCG DNA. The measured binding parameters are K_D_ = 1.20 μM and N = 0.48. The sequence of the CG duplex DNA used in the ITC binding study is listed above each panel. (**C**) ITC measurements of the binding of the SUVH5 SRA domain to the fully-5 hmCG DNA. The measured binding parameters are K_D_ = 1.50 μM and N = 0.52. (**D**) ITC measurements of the binding of the SUVH5 SRA domain to the hemi-5 hmCG DNA. The measured K_D_ = 7.6 μM. (**E**) ITC measurements of the binding of the SUVH5 SRA domain to the fully-5 caCG DNA. The measured K_D_ ~ 150.0 μM. (**F**) SUVH5 SRA binding to fully-5 fCG is endothermic. The measured K_D_ ~ 125.0 μM.

Here, we used an isothermal titration calorimetry (ITC) approach to investigate the K_D_ and stoichiometry for the binding of the SUVH5 SRA domain to the fully-5 hmCG duplex DNA. The SUVH5 SRA domain binds to fully-5 hmCG and fully-5 mCG with an affinity of approximately 1.0 μM, with two SRA molecules bound to a single fully-5 hmCG DNA duplex, as previously reported for fully-5 mCG (Fig. 1B,C)^4^. These binding studies demonstrate that SUVH5 SRA exhibits a similar stoichiometry and binding affinity for fully-5 hmCG- and fully-5 mCG-containing duplex DNAs. The affinity of SUVH5 SRA towards hemi-5 hmCG was ~7.6 μM (Fig. 1D), similar to what has been reported for hemi-5 mCG (5.0 μM)^4^, thereby confirming the preference of SUVH5 SRA for fully-5 mCG/5 hmCG over the hemi-modification. The thermodynamic parameters for 5 hmC-containing DNA binding to SUVH5 SRA are provided in Table 1.

### SUVH5 SRA exhibits reduced affinity for fully-5 fCG and fully-5 caCG DNA

We performed *in vitro* binding studies to investigate whether SUVH5 SRA recognizes the 5 hmC oxidation derivative bases 5 caC and 5 fC. Surprisingly, SUVH5 SRA exhibits only 150.00 μM binding affinity for fully-5 caCG DNA (Fig. 1E). SUVH5 SRA binding to fully-5 caCG is approximately 125- and 100-fold weaker compared with the binding to fully-5 mCG and fully-5 hmCG, respectively. Surprisingly, ITC titration of the binding of fully-5 fCG to SUVH5 SRA displayed an endothermic heat reaction and exhibited only 125.00 μM binding affinity (Fig. 1F). The thermodynamic data for the binding of 5 fC- and 5 caC-containing DNAs to SUVH5 SRA are listed in Table 1.

### Structure of SUVH5 SRA bound to fully-5 hmCG DNA

**Figure 2:**
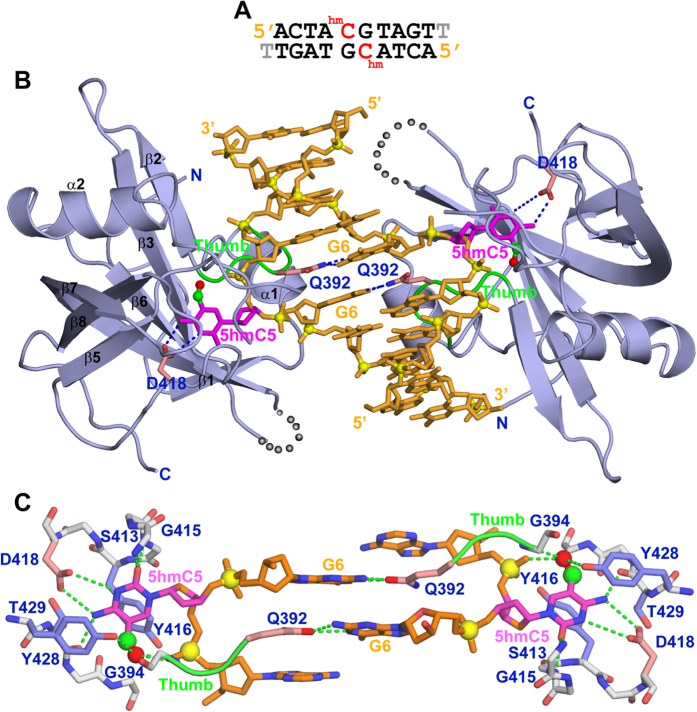
Crystal structure of the SUVH5 SRA domain bound to the fully-5 hmCG DNA. (**A**) Sequence of the 10-mer self-complementary fully-5 hmCG DNA (with a 3′-T overhang) containing 5 hmC-G base pairs in the centre of the duplex. (**B**) Stick (DNA) and ribbon (protein) representation of the 2.6 Å crystal structure of the 2:1 SUVH5 SRA-5 hmCG DNA duplex complex. The DNA is shown in orange, except for 5 hmC5, which is shown in magenta. The methylene and hydroxyl groups are shown as small green and red spheres, respectively, in Figs 2, 3 and 5. The backbone phosphorus atoms are shown as yellow balls, and the 5′ and 3′ ends of the DNA are labelled. The SRA domain is shown in blue, with its secondary structural elements labelled with the same α/β numbering scheme as that of the UHRF1 SRA^10^. The thumb loop is in green. The 5 hmC5s residues on the partner strands are flipped out of the minor groove and are positioned in the binding pockets of the individual SRA domains. The Watson-Crick edge of 5 hmC5 is hydrogen bonded with the side chain of Asp418. The side chain of Gln392 inserts into and fills the gap created by the flipped-out 5 hmC base and pairs with the Watson-Crick edge of the G6 base. (**C**) Magnified view of the relative alignments of the G6-Gln392 interactions and the interaction of the flipped-out symmetrical 5 hmC5s with the residues lining the binding pocket. Gln392 from the thumb loop is inserted into and fills the gap created by the flipped-out 5 hmC base, and its side chain forms stacking interactions with the flanking bases. The 5 hmC5 base is positioned between the aromatic rings of Tyr416 and Tyr428; its Watson-Crick edge is hydrogen bonded to the protein backbone and side chain of Asp418. The hydroxyl group at fifth position of 5 hmC forms intermolecular interactions with the side chain of Tyr428 and an amide group of G394 from the thumb and intramolecular interactions with the phosphate group.

The biochemical investigations described above raised an exciting possibility that SUVH5 SRA is also a 5 hmC “reader”. To understand the molecular mechanisms of SUVH5 SRA-mediated recognition of fully-5 hmCG DNA, we determined the crystal structure of SUVH5 SRA in a complex with a self-complementary 10-base pair duplex containing centrally located 5 hmC-G pairs with a 3′ “T” overhang (Fig. 2A). The biological assembly of the complex, two SRA molecules bound to duplex DNA, is generated by the crystallographic two-fold axis perpendicular to the DNA helical-axis (Fig. 2B). The symmetrical 5 hmC bases from the adjacent base pairs are flipped out and positioned in the binding pocket of individual SRA molecules (Fig. 2B). Gln392, located on the thumb loop (connects α1-helix and β2-strand), inserts into the minor groove and substitutes for the flipped out 5 hmC in the duplex DNA (Fig. 2B). In addition, Gln392 forms intermolecular hydrogen bonds with the Watson-Crick edge of the orphan guanine and stacking interactions with the flanking bases, thereby compensating for the extrusion of the 5 hmC bases from the DNA double helix (Fig. 2B,C).

### Recognition of the flipped out 5 hmC base in the SUVH5 SRA pocket

**Figure 3:**
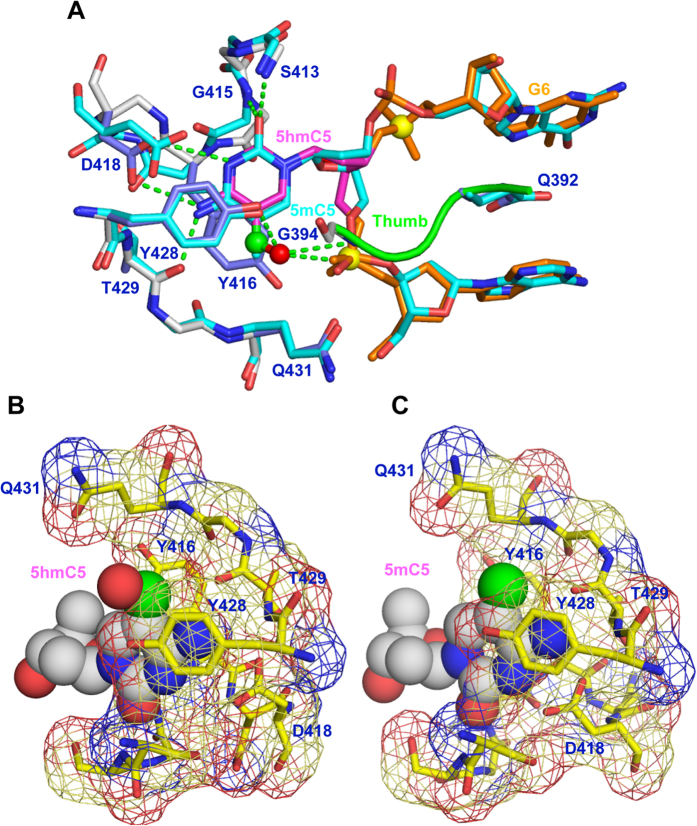
Comparison of the 5 hmC and 5 mC interactions in the SUVH5 SRA binding pocket. (**A**) Superposition of the flipped-out 5 hmC (magenta) and the flipped-out 5 mC (cyan) within their respective binding pockets in the structures of SUVH5 SRA-fully-5 hmCG and SUVH5 SRA-fully-5 mCG, respectively. (**B**) Accommodation of the flipped-out 5 hmC5 (space filling representation) in the SUVH5 SRA binding pocket (mesh with stick representation). (**C**) Accommodation of the flipped-out 5 mC5 (space filling representation) in the SUVH5 SRA binding pocket (mesh with stick representation).

The flipped out 5 hmCs are tightly positioned in the pocket via parallel-displaced π-stacking interactions with Tyr416 and Tyr428, and by intermolecular hydrogen bonds between its Watson-Crick edge and the side chain of Asp418. The walls of the pocket are primarily formed from the β3 to β5 strands (Fig. 2B,C). The main chain amide nitrogens and carbonyl oxygen of the wall form hydrogen bond interactions with O2 and N4 atoms of the 5 hmC. The hydroxyl group of 5 hmC participates in intermolecular interactions with the side chain of Tyr428 and the main chain nitrogen of Gly394 from the thumb loop. The hydroxyl group also has an intramolecular interaction with the phosphate group of 5 hmC (Figs 2C and 3A).

## Discussion

SUVH5 is unusual among the SRA domain proteins, as its SRA domain efficiently binds methylated and hydroxymethylated CG DNA, as well both the hemi- and fully modification statuses. Here, we discuss the mechanism by which the 5 mC oxidation derivative bases are recognized by the SRA domain proteins and the possible implications for epigenetic mechanisms.

### Comparison of 5 hmC and 5 mC recognition by the SUVH5 SRA

It was intriguing to determine how SUVH5 SRA recognizes 5 hmC in the binding pocket without losing affinity compared with 5 mC. SUVH5 SRA is the only “reader” whose structure in a complex with DNAs containing 5 mC^4^ and 5 hmC (current study) marks is known. Our structural analysis revealed that the flipped-out 5 hmC adopts a similar conformation in the binding pocket and has a similar distribution of intermolecular interactions as that of the flipped-out 5 mC in the crystal structure of the SUVH5 SRA-fully-5 mCG complex^4^ (Fig. 3A). 5 hmC binding induces a conformational change in the pocket, and the wall region (Gly414-Asp418) is reorganized to accommodate bases larger than 5 mC (Fig. 3A). Therefore, the SUVH5 SRA binding pocket accommodates both 5 hmC and 5 mC bases, without a significant change in the binding affinity (Figs 1B,C and 3).

The penalty for accommodating a larger base, 5 hmC, in the binding pocket may be compensated by the hydroxyl group specific interactions (Fig. 3A), thereby accounting for the insignificant difference in the binding affinity between fully-5 mCG and fully-5 hmCG for SUVH5 SRA (Fig. 1B,C). The methyl and hydroxymethyl functional groups are recognized differently by the SUVH5 SRA domain. The methyl group of 5 mC participates in van der Waals and hydrophobic contacts^4^, whereas the hydroxyl group of 5 hmC establishes both inter- and intramolecular polar interactions (Fig. 3).

### Comparison of the structures of the SUVH5 and UHRF2 SRA domains bound to 5 hmC-containing duplex DNA

**Figure 4:**
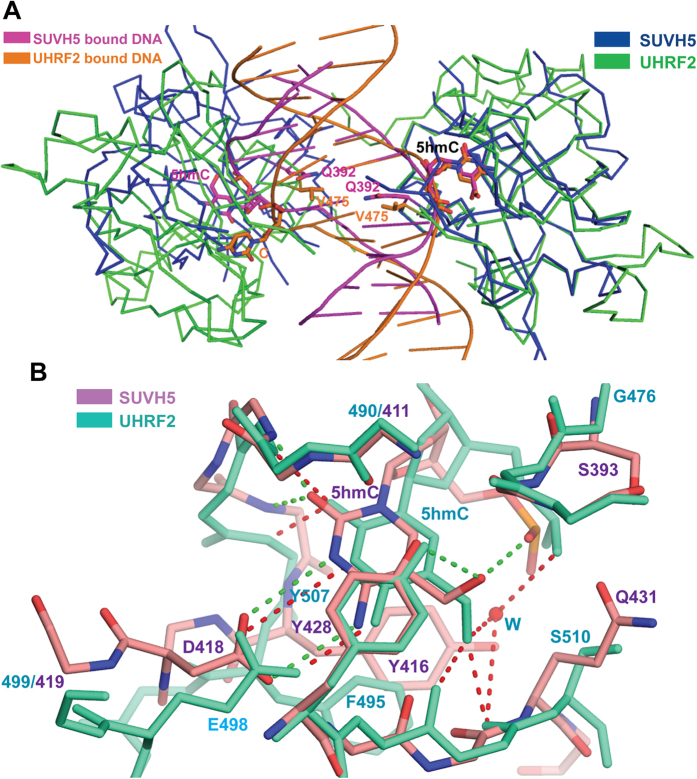
Structural comparison between the SRA domains of SUVH5 and UHRF2, showing the dual flip out and 5 hmC recognition. (**A**) Superposition of the complexes containing the SUVH5 SRA (blue ribbon) bound to the fully-5 hmCG DNA (magenta cartoon) onto UHRF2 SRA (green ribbon) bound to the hemi-5 hmCG DNA (orange cartoon). The SRA domains on the right are superimposed to allow the visualization of the relative displacement and orientation of the SRA domains on the left. (**B**) Superposition of the flipped-out 5 hmCs in the SUVH5 SRA (5 hmC and the binding pocket residues are shown as orange in atomic color) and UHRF2 SRA (5 hmC and the binding pocket residues are shown in greenish cyan) complexes within their respective binding pockets. The 5 hmC-mediated network of interactions with the binding pocket residues of SUVH5 SRA and UHRF2 SRA are depicted as dashed lines in green and red, respectively. A bridging water molecule is involved in the inter- and intramolecular recognition of the OH group of 5 hmC in UHRF2 SRA and is shown as a red sphere.

A notable difference in the recognition of 5 hmCG-containing DNA by the SRA domains of SUVH5 and UHRF2 is that the relative orientations of the two SRA domains are significantly altered upon binding to DNA (Fig. 4A). A comparative structural analysis also explains the basis for the preferential binding of UHRF2 to 5 hmC over 5 mC and the lack of preference exhibited by SUVH5. The loop segment that connects the β3-β5 strands, spanning residues 490–499 (411–419 in SUVH5) that forms one side of the binding pocket, moved away from the 5 hmC in UHRF2 SRA compared with the corresponding segment in SUVH5 SRA. This segment is composed of 10 residues in UHRF2 and 9 residues in SUVH5. Thus, a single residue insertion in UHRF2 leads to a significant increase in the pocket size in UHRF2 (Fig. 4B). Due to the movement in the loop segment, the negatively charged residue that interacts with the Watson-Crick edge of the flipped out 5 hmC is glutamate (Glu498) in UHRF2, whereas corresponding residue is aspartate (Asp418) in SUVH5. In addition, the residues that form the pocket in SUVH5 have bulkier side chains compared with the residues in UHRF2 at the corresponding positions (Ser393, Tyr416 and Gln431 in SUVH5 correspond to Gly476, Phe495 and Ser510 in UHRF2) (Fig. 4B). In conclusion, the subtle sequence and structural variations in the binding pockets of the SRA domains lead to preferential recognition of different marks by the SUVH5 and UHRF2 proteins.

### Comparison of the DNA and flipped base recognition by SUVH5 SRA with the SRA-like domain of prokaryotic endonuclease

The SRA-like domains of endonucleases, MspJI^23^, PvuRts1I^21^, LpnPI^24^ and AspBHIa^25^, in prokaryotes recognize 5 hmC and/or 5 mC in various sequence contexts. The overall structure of the SRA-like domain of MspJI is similar to that of the SUVH5 SRA domain (RMSD 2.53 Å over 123 Ca atoms). The SRA-like domain of MspJI employs a base-flipping mechanism to recognize the 5 mC base in the 5′-5 mCNNR-3′ (N is any nucleotide and R is A or G) sequence context, which is reminiscent of 5 mC recognition by the SUVH5 SRA domain (Supplementary Fig. S1 online). The base flipping promotion loop, Loop-B3, of MspJI approaches the minor groove of the DNA to recognize 5 mC (Supplementary Fig. S1B online). Loop-B3 is structurally and functionally equivalent to the thumb loop of SUVH5 SRA. Both loops provide a residue that substitutes for 5 mC or 5 hmC (Gln392 in SUVH5 and Glu65 in MspJI) in the duplex DNA and pairs with the orphaned guanine (Supplementary Fig. S1 online). Notable differences in recognition of the DNA by SUVH5 and MspJI are: (A) two molecules of SUVH5 SRA recognize the fully-5 hmCG DNA, in contrast to a single molecule of the SRA-like domain of MspJI recognizes 5 mC containing DNA; (B) MspJI engages an additional loop, Loop-2B. Loop-2B also interrogates the DNA at the minor groove through the Gln33 residue (Supplementary Fig. S1 online).

The recognition of the flipped-out base by the SUVH5 and MspJI proteins is highly similar. Both proteins recognize the base through π-stacking interactions with the aromatic residues in the binding pocket. In addition, the Watson-Crick edge of 5 mC or 5 hmC participates in hydrogen bonds with aspartic acid and the main chain atoms of the amino acids in the binding pocket (Supplementary Fig. S1 online)

### Basis for the weaker binding of SUVH5 SRA to 5 fC- and 5 caC-containing DNAs

**Figure 5:**
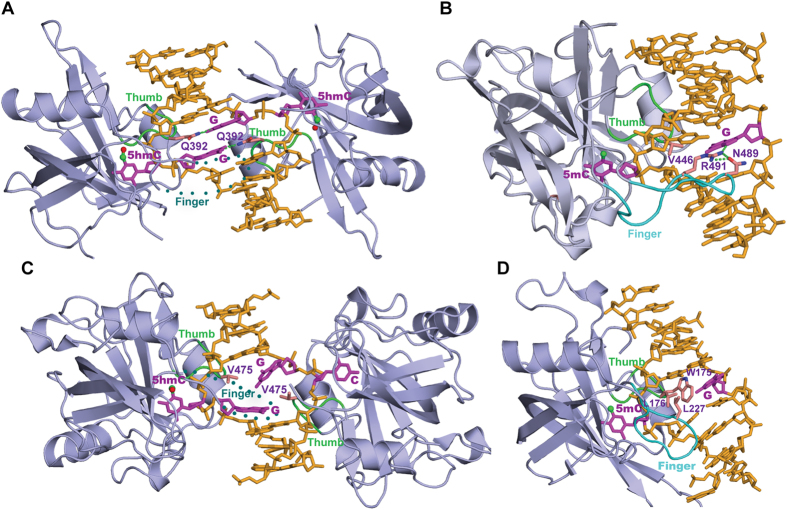
Structural comparison between the SRA domains of SUVH5, UHRF1, UHRF2 and SUVH4 bound to the fully-5 hmCG, hemi-5 mCG, hemi-5 hmCG and hemi-5 mCHG duplex DNA. (**A**) Structure of the 2:1 SUVH5 SRA-fully-5 hmCG DNA duplex complex. Note the dual flip-out of the symmetrical 5 hmCs from adjacent base pairs on the partner strands. The thumb loop, which is involved in 5 hmC flipping, and the disordered finger segment are shown in green and dotted cyan, respectively. For clarity, only the finger loop from the left SRA is indicated. (**B**) Structure of the 1:1 UHRF1 SRA-hemi-5 mCG DNA duplex complex, where only 5 mC is flipped out from the duplex DNA (PDB: 3CL2) and is recognized by the single SRA domain. Two loops, finger and thumb, are shown in green and cyan, respectively. The residues on the finger loop partially fill the hole left by the flipped out 5 mC base, and functions by interacting with the orphaned G and could shield the unmodified C from being recognized by the second SRA domain. (**C**) Crystal structure of the 2 UHRF2 SRA domains bound to a hemi-5 hmCG DNA (PDB: 4PW6). 5 hmC and C are flipped out from adjacent base pairs on the partner strands and are positioned in the binding pockets of the individual SRA domains. The mode of recognition is similar to SUVH5 SRA bound to fully-5 hmCG. The thumb and disordered finger loops are indicated as described for the structure of the SUVH5-fully-5 hmCG complex shown in Fig. 5A. (**D**) Structure of an SUVH4 SRA domain bound to the hemi-5 mCHG duplex DNA (PDB: 4QEP). Only 5 mC is flipped out from the duplex DNA and is positioned in the SRA binding pocket. The thumb and finger loops are indicated as described for the structure of the UHRF1-hemi-5 mCG complex. Both the thumb and finger loops, which are involved in base flipping, interrogate the 5 mCHG duplex DNA through minor and major grooves, respectively. Leucine residues from the thumb and finger loops fill the holes left by the flipped out 5 mC and are involved in van der Waals interactions within the duplex DNA.

Unlike 5 hmC recognition, SUVH5 SRA exhibits significantly weaker affinity for both 5 fC- and 5 caC-containing duplex DNAs (Fig. 1E,F). However, the modelled 5 caC and 5 fC bases fit very well in the binding pocket of SUVH5 SRA (data not shown). The proteins involved in base flipping rely on remarkably specific detection mechanisms to locate the modified or damaged base in the duplex DNA. The base flipping mechanisms might involve two sequential steps. In the first step, the protein can probe and detect the modified base in the genomic DNA. In the second step, modified base in the duplex DNA is substituted by the amino acid, followed by the insertion of flipped out base into their binding or active site pockets^26^,^27^. We hypothesize that the SRA domain may be unable to complete the first step in 5 caC or 5 fC base recognition. Our hypothesis is supported by the structure of the 5 fC-containing DNA double helix, where 5 fC alters the geometry of the grooves and base pairs associated with the modified base, including those leading to helical under-winding and the narrowing of the major groove while opening the minor groove^28^. The SRA domains of SUVH5 (Fig. 5A), UHRF1 (Fig. 5B), UHRF2 (Fig. 5C) and SUVH4 (Fig. 5D) interrogate the minor groove of B-form DNA using the thumb loop to flip out the modified cytosine. In conclusion, CG formylation induced topological changes in the duplex DNA structure, particularly in the minor groove, which could negatively affect the recognition of 5 fC by the SRA domains. However, further studies are required to understand the reasons that the binding of SUVH5 SRA to fully-5 caCG is driven by enthalpy, whereas the binding to fully-5 fCG is driven by entropy.

### The finger loop determines dual or single flip-out of the 5 mC- or 5 hmC-modified bases by the SRA domain proteins

It is intriguing that the SRA domains of UHRF1 and UHRF2 selectively recognize hemi-5 mCG and fully-5 hmCG, respectively (Fig. 5B,C)^18^, even though they have 88% sequence similarity and high structural identity (RMSD: 0.73 Å). Similarly, the SRA domains of SUVH5 and SUVH4 (aka KRYPTONITE) from *Arabidopsis* have high structural (RMSD: 1.14 Å) and sequence (67%) similarities; however, the former prefers fully-status (fully-5 mCG/fully-5 hmCG) (Figs 4A and 5A), whereas the latter exhibits selectivity for the hemi-status (hemi-5 mCHG or 5 mCHH) (Fig. 5D)^4^,^19^. The structures of the SRA domains from the aforementioned protein modules in complex with the cognate DNA have revealed the basis for this selectivity (Fig. 5). Hemi-status recognition by the SRA domains of UHRF1 and SUVH4 is correlated with the engagement of the finger loop that facilitates the flipping of the modified C (5 mC) only, and it could shield the unmodified C from being recognized by the second SRA domain (Fig. 5B,D)^10^,^19^. Additionally, in both the cases, it interrogates the duplex DNA through the major groove (Fig. 5B,D). By contrast, the finger loop is disordered in the preferential fully-5 mCG or fully-5 hmCG binders, such as SUVH5 and UHRF2. These binders only rely on the thumb loop, and a residue present in this loop inserts into the DNA through the minor groove and substitutes for the modified (5 mC or 5 hmC) or unmodified “C” located on the partner strands of the duplex DNA (Fig. 5A,C)^4^,^18^. Therefore, these modules recognize both modification (hemi and fully) statuses through a dual flip-out of the bases^4^,^18^. Taken together, our analyses provide the structural basis for the selectivity of the DNA modification status (hemi or fully) by the SRA domains of protein modules from plants and mammals.

### Possible implications of 5 mC oxidation in 5 mC mark interpretation

Until recently, 5 mC was considered the only epigenetic mark in genomic DNA. However, newly identified marks either antagonize the read-out and interpretation of 5 mC or they can act as new set of epigenetic marks that are recognized by different epigenetic reader modules^29^. Conversely, UHRF2, a close relative of UHRF1, specifically binds 5 hmC in neuronal progenitor cells^29^. Recently, it has been shown that the oxidation of 5 mC to 5 hmC clearly interferes with the DNA binding to the MBD domain of MeCP2, MBD1 and MBD2^30^,^31^. Similarly, the current study indicates that the SRA domains of UHRF1 (Supplementary Fig. S2 online) and SUVH5 exhibit lower binding affinity for 5 fC- and 5 caC-containing DNAs. As these proteins are involved in transcriptional repression through 5 mC recognition, the oxidation of 5 mC to 5 caC could promote a switch from a repressive to an active transcriptional state of the chromatin, thereby changing the cellular interpretation of the 5 mC epigenetic mark.

UHRF1 binds to both hemi-5 mCG and DNA methyltransferase 1 (DNMT1) to maintain the DNA methylation patterns in mammals^7^,^8^,^10^. Similarly, SUVH5 and SUVH4, in coordination with a DNA methyltransferase CMT3, are involved in DNA and H3K9 methylation through the recognition of a 5 mC mark by the SRA domain in plants^3^. We speculate that UHRF1 and SUVH5 do not facilitate DNA methylation maintenance through the aforementioned mechanisms in the presence of 5 fC and 5 caC bases in the genome, because the SRA domains of these proteins bind weakly to these bases (Supplementary Fig. S2 online and Fig. 1E,F).

Recent studies have not only demonstrated the widespread existence of 5 hmC, 5 fC and 5 caC in the genomic DNA of various plant species and tissues^15^,^32^, but also unearthed their role in the regulation of gene expression during drought or salt stress^15^,^33^,^34^. Environmental stresses such as drought and salinity could also change the content of the 5 fC and 5 caC bases^15^. We speculate that the discrimination in the recognition of 5 mC oxidation derivatives by SUVH family proteins in different plant tissues and in response to environmental stresses may have a role in epigenetic regulation.

## Materials and Methods

### Protein purification

The expression and sequential purification of SUVH5 SRA were performed as previously described^4^. The hexahistidine-sumo-tagged construct containing SUVH5 SRA (residues 362–528) was expressed in *Escherichia coli* Rosetta2 DE3. The expressed protein was purified on a nickel-charged column (HisTrap HP, GE Healthcare). The fusion protein was cleaved with 15 U mL^−1^ of Ulp1 protease. The protein was further purified by cation-exchange (HiTrap Heparin HP) chromatography. Gel filtration chromatography was used as the final purification step. The protein was purified using a gel filtration column (HiLoad Superdex 200 26/60), which was equilibrated with a buffer containing 15 mM Tris-HCl, pH 7.5, 100 mM NaCl, 3 mM DTT and 2.5% Glycerol. The purified protein was concentrated to 15 mg mL^−1^ at 4 °C in Vivaspin 20 mL (Vivascience AG) 10,000 cut-off concentrator.

### DNA preparation

The modified (containing a central 5 mC, 5 hmC, 5 fC or 5 caC base) or unmodified DNA sequences were dissolved in buffer containing 25 mM Tris-HCl pH 7.5, 25 mM MgCl_2_ and 75 mM NaCl. The fully-5 mCG, fully-5 hmCG, fully-5 fCG and fully-5 caCG duplexes were generated by heating the self-complementary single strand DNA sequences containing the centrally located modified base to 95 °C for 5 minutes and then cooling on ice for 5 hours. Similarly, to generate the hemi-5 hmCG DNA duplex, the complementary strands (5 hmC-containing and unmodified) were mixed in an equimolar ratio and annealed as described above.

### Isothermal Titration Calorimetry (ITC) measurements

The equilibrium dissociation constant (K_D_), molar ratio (N) and thermodynamic parameters of the SUVH5 SRA domain bound to fully-5 mCG, fully-5 hmCG, hemi-5 hmCG, fully-5 fCG or fully-5 caCG were determined using a VP-ITC calorimeter (MicroCal, LLC) at 25 °C.

The protein and duplex DNA were dialysed against a buffer containing 40 mM Tris-HCl, 50 mM NaCl, and 2 mM β-mercaptoethanol, pH 7.5, overnight at 4 °C. The protein and duplex DNA concentrations used were 100 μM to 150 μM and 0.5 mM to 0.75 mM, respectively, for the fully-5 mCG, fully-5 hmCG and hemi-5 hmCG binding studies. For the fully-5 fCG and fully-5 caCG binding studies, the concentrations of both the protein and DNA were increased by 3-fold. The volume of SUVH5 SRA domain in the reaction cell was 200 μL, and the reference cell was filled with deionized water. The modified duplex DNA was sequentially added in 2.3 μL (for a total of 15–16 injections) aliquots at 3-min intervals. The data were processed using MicroCal Origin software. The titration data were deconvoluted based on a binding model containing “One set of sites” using a nonlinear least-squares algorithm. The binding enthalpy change (ΔH), association constant (Ka), and binding stoichiometry (N) were permitted to vary during the least-squares minimization process and taken as the best-fit values for SUVH5 SRA domain bound to the fully-5 mCG, fully-5 hmCG or hemi-5 hmCG DNA. In the cases where the SUVH5 SRA domain was bound to fully-5 caCG and/or fully-5 fCG, ‘N’ was fixed to 0.5, and ‘Ka’ and ‘ΔH’ were permitted to float. The reported values are the best values from three titrations. The errors reported by the program are shown as standard deviations.

### Crystallization

The crystals were grown using 5 mg mL^−1^ of protein in a protein to duplex DNA molar ratio of 1:0.6. All crystals were grown at 18 °C using the sitting-drop method by mixing 150 nL of the protein solution with 150 nL of the well solution using the Mosquito crystallization robot. The crystals of the SUVH5 SRA-fully-5 hmCG complex were grown from a condition containing 0.2 M Sodium chloride, 0.1 M BIS-TRIS pH 6.5, and 25% w/v Polyethylene glycol 3,350. The crystals were flash frozen at 100 K in a cryoprotectant containing mother liquor and 12% ethylene glycol.

### Crystal data collection, structure determination and refinement

The diffraction data of the crystals of the SUVH5 SRA-fully-5 hmCG complex were collected at 100 K at the Brookhaven National Laboratory’s beam-line X29. The crystals were diffracted to 2.6 Å. The intensity integration, merging and scaling of the data were performed with HKL-2000. The crystals belong to space group P4_2_2_1_2, with unit cell dimensions of a = b = 76.98 Å and c = 72.11 Å.

The structure of the SUVH5 SRA-fully-5 hmCG DNA complex was solved by molecular replacement using MOLREP^35^ from the CCP4 suite^36^, using the SUVH5 SRA monomer (PDB: 3Q0B) as a search model. The asymmetric unit contains a single SRA molecule and a strand of self-complementary DNA. One strand of the self-complementary DNA was built into the density map using the COOT program^37^ and the structure of the complex was refined against the 2.6 Å diffraction data using simulated annealing, followed by automatic target function with X-ray/stereochemistry weight optimization refinement using the PHENIX program^38^. The final R-free and R-factor values of the model were 28.2% and 23.4%, respectively. The X-ray data collection and refinement statistics are listed in Table 2.

### Protein Data Bank entry

The X-ray coordinates and structure factors of the structure of the SUVH5 SRA-fully-5 hmCG complex have been deposited in the Protein Data Bank (PDB) with the accession code 4YGI.

## Additional Information

**How to cite this article**: Rajakumara, E. *et al*. Mechanistic insights into the recognition of 5-methylcytosine oxidation derivatives by the SUVH5 SRA domain. *Sci. Rep*. **6**, 20161; doi: 10.1038/srep20161 (2016).

## Supplementary Material

Supplementary Information

## Figures and Tables

**Table 1:** Dissociation constants and thermodynamic data for the binding of the modified deoxycytidine base-containing duplex DNAs to the SUVH5 SRA.

Modified Duplex DNA	K_D_ (μM)	ΔH (kcal/mol)	TΔS (kcal/mol)
Fully-5 mCG	1.20 ± 0.09	−30.7 ± 1.1	−22.4
Fully-5 hmCG	1.50 ± 0.12	−32.1 ± 1.1	−24.0
Hemi-5 hmCG	7.6 ± 0.6	−10.8 ± 0.5	−3.7
Fully-5 caCG	150 ± 14	−6.3 ± 0.2	−2.2
Fully-5 fCG	125 ± 15	3.2 ± 0.2	5.3

**Table 2:** Summary of the X-ray diffraction data and structure refinement statistics.

Crystal	SUVH5 SRA-fully-5 hmCG CG DNA
Beam Line	BNL-X29
Wavelength (Å)	1.0718
Space group	*P*4_2_2_1_2
Unit Cell	
a, b, c (Å)	76.98, 76.98, 72.11
Resolution (Å)	30-2.60 (2.69-2.60)^a^
R_sym_	0.05 (0.79)^a^
I/σ (I)	35.5 (2.4)^a^
Completeness (%)	98.8 (99.1)^a^
Redundancy	4.6 (4.1)^a^
Number of unique reflections	7060
R_work_/R_free_ (%)	23.4/28.2
Number of non-H atoms	
Protein	1133
DNA	204
Water	16
Magnesium	3
Average B factors (Å^2^)	
Protein	69.2
DNA	63.9
Water	68.1
Magnesium	73.9
R.M.S deviations	
Bond lengths (Å)	0.003
Bond angles (^o^)	0.61

^a^The value for the highest resolution shell is shown in parentheses.
