# Supplementary material for: Mechanistic insights into the recognition of 5-methylcytosine oxidation derivatives by the SUVH5 SRA domain
Source: Sci Rep. 2016 Feb 4;6:20161. doi: 10.1038/srep20161 (PMC4740795; doi:10.1038/srep20161)
Supplement: Supplementary Information [file srep20161-s1.pdf]

Supplementary information

Mechanistic insights into the recognition of 5-methylcytosine oxidation derivatives by the SUVH5 SRA domain

Eerappa Rajakumara [1,#], Naveen Kumar Nakarakanti [1,*], M. Angel Nivya [1,*] & Mutyala Satish [1]

1. Department of Biotechnology, Indian Institute of Technology Hyderabad, Kandi, Sangareddy, 502285, Telangana, India

* These authors are contributed equally to this work.

[#] Corresponding author address: Department of Biotechnology, Indian Institute of Technology Hyderabad, Kandi, Sangareddy, 502285, Telangana, India. Telephone No. +91 40 2301 7002. E-mail: eraj@iith.ac.in

Supplementary Figures S1-2 and Figure Legends

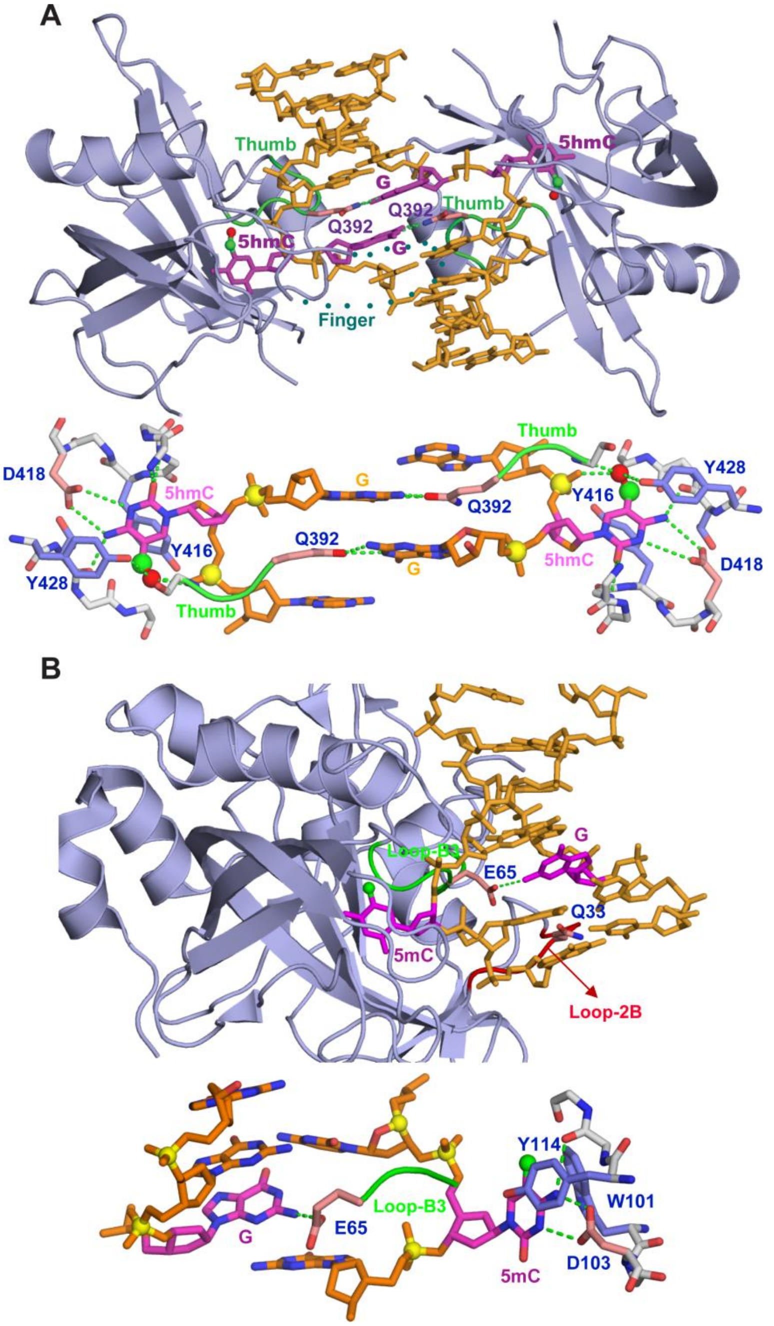

Supplementary Figure S1. DNA and flipped-out base recognition by the SRA domain of SUVH5 and the SRA-like domain of MspJI.

(A) Fully-5hmCG DNA recognition by the SUVH5 SRA. Upper panel: Two molecules of SRA recognize single fully-5hmCG DNA. Base flipping promotion loop, thumb, interrogates the DNA

through minor groove, and substitutes the 5hmC in the duplex DNA is colored in green. Side chain of Gln392 inserts into and fills the gap created by the flipped-out 5hmC base, and pairs with the orphaned G base. Lower panel: Recognition of 5hmC in the binding pocket. The 5hmC base is positioned between the aromatic rings of Tyr416 and Tyr428. Watson-Crick edge of 5hmC is hydrogen-bonded to the Asp418 side chain.

(B) 5mC containing DNA recognition by prokaryotic MspJI protein (PDB ID: 4R28). Upper panel: Single molecule of MspJI recognizes single methylated duplex DNA. Loop-B3 or 'base-flipping-promotion' loop (green), approaches the DNA from the minor grove, and substitutes the 5mC base in the duplex DNA. The side chain of Glu65 fills the gap created by the flipped-out 5mC base, and pairs with the orphaned G base in the duplex DNA. The Loop-2B is positioned in the minor groove of the flipped 5mC is colored in red. Lower panel: Recognition of 5mC in the binding pocket. 5mC is positioned between two aromatic residues Trp101 and Tyr114, and Asp103 is making hydrogen bond with Watson-Crick edge of the 5mC.

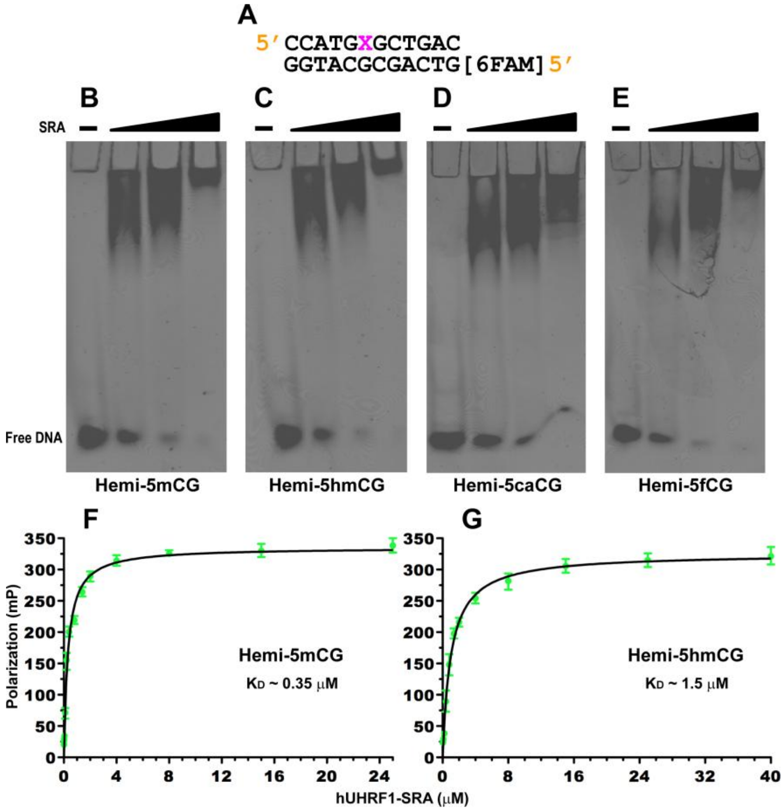

Supplementary Figure S2. Binding of the UHRF1 SRA domain to hemi-5mCG, hemi-5hmCG, hemi-5caCG and hemi-5fCG containing duplex DNAs.

(A) Sequence of the 5’-6FAM labeled 12 mer duplex DNA used for EMSA and FP studies. ‘X’ is 5mC, 5hmC, 5caC or 5fC base.

(B) EMSA binding study of UHRF1 SRA with hemi-5mCG DNA. (_) indicates no protein but only DNA. Un-bound (free) DNA is indicated.

(C) EMSA binding study of UHRF1 SRA with hemi-5hmCG DNA.

(D) EMSA binding study of UHRF1 SRA with hemi-5caCG DNA.

(E) EMSA binding study of UHRF1 SRA with hemi-5fCG DNA.

(F) Fluorescence Polarization (FP) measurements of the binding of the UHRF1 SRA domain to hemi-5mCG DNA. The measured K_D = 0.35 µM.

(G) FP measurements of the binding of the UHRF1 SRA domain to hemi-5hmCG DNA. The measured K_D = 1.5 µM.

Supplementary Results

EMSA and FP studies of the UHRF1 SRA with hemi-5mCG, hemi-5hmCG, hemi-5fCG and hemi-5caCG duplex DNAs

We attempted to determine the K_D for binding of 5mC oxidation derivative bases in hemi-CG sequence context towards UHRF1 SRA through ITC measurements, however there was negligible heat change during ITC titration. Therefore, we decided to employ Electrophoretic Mobility Shift Assay (EMSA) and Fluorescence Polarization (FP) techniques.

Although UHRF1 SRA binding to hemi-5mCG[1–4] and hemi-5hmCG[5,6] was reported, no data is available on its ability to recognize hemi-5caCG and hemi-5fCG modifications. Here we performed EMSA, using 5'-6FAM labeled DNA duplexes in a CG sequence context (Supplementary Figure S2A), to investigate the UHRF1 SRA specificity for hemi-5caCG and hemi-5fCG DNA duplexes. Our control EMSA experiments revealed that UHRF1 SRA binds to hemi-5mCG (Supplementary Figure S2B) and hemi-5hmCG (Supplementary Figure S2C) DNA similar to published data[3,5]. Though UHRF1 SRA could also recognize hemi-5caCG and hemi-fCG, concentration of protein and modified DNA used for binding study is more than that used for 5hmC owing to weaker binding affinity towards 5caC and 5fC containing DNAs (Supplementary Figure S2D and E). Furthermore, our finding showed that the SRA domain of UHRF1 binds to all 5mC oxidation derivatives.

To quantify the UHRF1 SRA binding affinity for 5mC oxidation derivatives in hemi-CG sequence context, we performed FP measurements using 5'-6FAM labeled DNA duplexes (Supplementary Figure S2A). UHRF1 SRA domain binds to hemi-5mCG DNA with a K_D of 0.35 µM (Supplementary Figure S2F) which is similar (0.20 µM) to reported in literature[7]. However, the binding constant decreases by a factor of 4.3 (K_D = 1.5 µM) for hemi-5hmCG (Supplementary Figure S2G). Our preliminary FP study suggests that UHRF1 SRA binds to hemi-5caCG and hemi-5fCG with much higher K_D values (weaker affinity) when compared to hemi-5hmCG. However, obtaining data points at higher concentration of protein and subsequent

K_D estimation was unsuccessful as protein was precipitating at higher concentration when it mixed with the hemi-5caCG or hemi-5fCG DNA duplex.

Supplementary Materials and methods

UHRF1 SRA Purification

Expression and sequential purification of UHRF1 SRA was performed as described earlier[8]. Hexahistidine-sumo tagged construct containing UHRF1 SRA (residues 427-630) was expressed in *Escherichia coli* Rosetta2 DE3. Expressed protein was purified on a nickel-charged column (HisTrap HP, GE healthcare). Fusion protein was cleaved with Ulp1 protease at 15 UmL^{-1}. The protein was further purified by cation-exchange (HiTrap Heparin HP) chromatography. Finally, protein was purified using gel filtration chromatography using a 'HiLoad Superdex 200 26/60' column, which was equilibrated with a buffer containing 15 mM Tris-HCl, pH 7.5, 100 mM NaCl, 3 mM DTT and 2.5% Glycerol. Purified protein was concentrated to 15 mg mL^{-1} at 4 ºC in Vivaspin 20 mL (Vivascience AG) 10,000 cut-off concentrator.

Electrophoretic Mobility Shift Assay

Binding between the UHRF1 SRA domain and 5'-6FAM-labeled duplex DNA was carried out in a buffer containing 25 mM Tris-HCl, pH 7.5, 5% Glycerol, 60 mM NaCl, 10 mM MgCl2. 0.4 mg/mL BSA and 2 mM dithiothreitol with increasing amounts of protein (200 to 1000 pmol for hemi-5mCG and hemi-5hmCG binding study and, 600 pmol to 3 nmol for hemi-5fCG and hemi-5caCG DNA) and fixed amount of modified DNA duplex (3 μg of hemi-5mCG and hemi-5hmCG DNA, and 9 μg of hemi-5fCG and hemi-5caCG DNA). The 20 μL of reaction mixtures were incubated at 4°C for 45 minutes then electrophoresed on a native 6% polyacrylamide gel at 100 V for 1.0 hour in a buffer containing 100 mM Tris-borate-EDTA and 2.5% glycerol in the cold room. The gels were fluorescence scanned using Typhoon TRIO Variable Mode Imager.

Fluorescence Polarization measurements

Polarization measurements of UHRF1 SRA domain binding to 5'-6FAM labeled duplex DNA was performed at 20 °C. The 5'-6FAM-labeled duplex DNA (50 nM) was added to a UHRF1 SRA domain with increasing concentration (50 nM to 50 μM) of protein. The polarization (in millipolarization [mP] units) of 20 μL of reaction mixture was measured after 45 minutes of incubation at room temperature. Polarization values were referenced against a blank sample buffer (20 mM Tris-HCl pH 7.5, 50 mM NaCl and 1 mM DTT) and a reference contained only 50 nM DNA. The polarization data were analyzed using GraphPad Prism 5.0 software (GraphPad, San Diego, CA, USA) to calculate a binding dissociation constant (K_D) by fitting the experimental data in a non-linear regression equation using a one-site specific binding model accounting for ligand depletion. Experiments were performed in triplicate.

References:

1. Arita, K., Ariyoshi, M., Tochio, H., Nakamura, Y. & Shirakawa, M. Recognition of hemi-methylated DNA by the SRA protein UHRF1 by a base-flipping mechanism. *Nature* **455,** 818–821 (2008).
2. Avvakumov, G. V. *et al.* Structural basis for recognition of hemi-methylated DNA by the SRA domain of human UHRF1. *Nature* **455,** 822–825 (2008).
3. Bostick, M. *et al.* UHRF1 plays a role in maintaining DNA methylation in mammalian cells. *Science* **317,** 1760–1764 (2007).
4. Hashimoto, H. *et al.* The SRA domain of UHRF1 flips 5-methylcytosine out of the DNA helix. *Nature* **455,** 826–829 (2008).
5. Frauer, C. *et al.* Recognition of 5-hydroxymethylcytosine by the Uhrf1 SRA domain. *PloS One* **6,** e21306 (2011).
6. Hashimoto, H. *et al.* Recognition and potential mechanisms for replication and erasure of cytosine hydroxymethylation. *Nucleic Acids Res.* **40,** 4841–4849 (2012).

7. Qian, C. *et al.* Structure and hemimethylated CpG binding of the SRA domain from human UHRF1. *J. Biol. Chem.* **283,** 34490–34494 (2008).
8. Rajakumara, E. *et al.* PHD finger recognition of unmodified histone H3R2 links UHRF1 to regulation of euchromatic gene expression. *Mol. Cell* **43,** 275–284 (2011).
